# Fondaparinux versus Enoxaparin - Which is the Best Anticoagulant for Acute Coronary Syndrome? - Brazilian Registry Data

**DOI:** 10.5935/abc.20160127

**Published:** 2016-09

**Authors:** Alexandre de Matos Soeiro, Pedro Gabriel Melo de Barros e Silva, Eduardo Alberto de Castro Roque, Aline Siqueira Bossa, Maria Cristina César, Sheila Aparecida Simões, Mariana Yumi Okada, Tatiana de Carvalho Andreucci Torres Leal, Fátima Cristina Monteiro Pedroti, Múcio Tavares de Oliveira Jr.

**Affiliations:** 1 Unidade Clínica de Emergência - Instituto do Coração (InCor) do Hospital das Clínicas da Universidade de São Paulo - Brazil; 2 Hospital TotalCor, São Paulo, SP - Brazil; 3 Hospital Metropolitano, Serra, ES - Brazil

**Keywords:** Acute Coronary Syndrome, Anticoagulants / therapeutic use, Enoxaparin / therapeutic use, Myocardial Infarction, Percutaneous Coronary Intervention, Hemorrhage

## Abstract

**Background:**

Recent studies have shown fondaparinux's superiority over enoxaparin in patients with non-ST elevation acute coronary syndrome (ACS), especially in relation to bleeding reduction. The description of this finding in a Brazilian registry has not yet been documented.

**Objective:**

To compare fondaparinux versus enoxaparin in in-hospital prognosis of non-ST elevation ACS.

**Methods:**

Multicenter retrospective observational study. A total of 2,282 patients were included (335 in the fondaparinux group, and 1,947 in the enoxaparin group) between May 2010 and May 2015. Demographic, medication intake and chosen coronary treatment data were obtained. Primary outcome was mortality from all causes. Secondary outcome was combined events (cardiogenic shock, reinfarction, death, stroke and bleeding). Comparison between the groups were done through Chi-Square test and T test. Multivariate analysis was done through logistic regression, with significance values defined as p < 0.05.

**Results:**

With regards to treatment, we observed the performance of a percutaneous coronary intervention in 40.2% in the fondaparinux group, and in 35.1% in the enoxaparin group (p = 0.13). In the multivariate analysis, we observed significant differences between fondaparinux and enoxaparin groups in relation to combined events (13.8% vs. 22%. OR = 2.93, p = 0.007) and bleeding (2.3% vs. 5.2%, OR = 4.55, p = 0.037), respectively.

**Conclusion:**

Similarly to recently published data in international literature, fondaparinux proved superior to enoxaparin for the Brazilian population, with significant reduction of combined events and bleeding.

## Introduction

The use of anticoagulant agents in ACS is essential, impacting on the reduction of events and mortality. However, the choice of a better anticoagulant therapy for patients with ACS is still controversial, and it is currently a widely discussed topic. Logic would state that, the more effective the anticoagulant, the higher the risk of bleeding and vice-versa.^1,2^

Recent studies have shown fondaparinux to be superior to enoxaparin for patients with non-ST elevation ACS (NSTEACS), especially in relation to bleeding.^3-5^ The description of this finding has yet to be documented in a Brazilian registry.

Thus, we have developed this study to compare fondaparinux to enoxaparin in in-hospital prognosis of NSTEACS in the Brazilian population.

## Methods

### Study Population

This is an observational multicenter retrospective study. A total of 2,282 patients with NSTEACS admitted between May 2010 and May 2015 in the emergency sector were included. Patients were divided into two groups: fondaparinux (N = 335) and enoxaparin (N = 1,947). ST elevation was the only exclusion criterion employed. All patients were submitted to a cineangiocardiography.

Presence of ACS was considered in all patients who met the established criteria on the latest guidelines from the Brazilian Society of Cardiology and the American Heart Association.^6,7^ Non-ST elevation ACS was defined as the presence of chest pains associated to electrocardiographic alterations or troponin elevation/drop during hospital stay, or, in the absence of those, clinical conditions and risk factors compatible with unstable angina (severe or progressive chest pains at rest or at minimum effort). Major bleeding was defined using the BARC score^8^ types 3 and 5, and minor bleeding using types 1 and 2. Reinfarction was considered in the presence of chest pain reoccurrence associated with a new troponin elevation. Ischemic stroke was considered in the presence of new motor focal neurological deficit confirmed by computerized tomography of the head. Patients on fondaparinux received an additional dose of unfractionated intravenous heparin when undergoing percutaneous coronary intervention (60 UI/kg when on glycoprotein IIb IIIa inhibitors, or 85 Ul/kg when patients were not on the medication).

The following data were obtained: age, gender, presence of diabetes mellitus, systemic arterial hypertension, smoking habit, dyslipidemia, family history of early onset coronary disease, previous coronary artery disease (previous angioplasty or coronary artery bypass surgery), hemoglobin, creatinine, peak troponin, Killip classification, left ventricle ejection fraction, medications used in the first 24 hours of hospital admission and adopted coronary treatment.

The study was submitted to and approved by the Research and Ethics Committee. Informed consent was filled out by all patients included in the study.

### Statistical Analysis

Primary outcome was in-hospital mortality from all causes. Secondary outcome was combined events (cardiogenic shock, myocardial infarction, death, ischemic stroke and major bleeding). Descriptive analysis was done using means, minimum and maximum values. Comparisons between groups were done using Chi-Square test for categorical variables. For continuous variables, when Kolmogorov-Smirnov normality test showed normal distribution, the t test was used, with significance considered at p < 0.05. When the distribution did not follow the normality pattern, we used the Mann-Whitney U test. Multivariate analysis was done by logistic regression, with significance considered at p < 0.05. We considered all basal characteristics presented in Table 1 as variables in the analysis.

**Table 1:** Basal clinical characteristics of patients on fondaparinux versus enoxaparin in the studied sample

	Fondaparinux	Enoxaparin	p
Age (mean)	61 ± 11.39	61.8 ± 10.45	0.25
Male (%)	65.7%	62.6%	0.228
Diabetes Mellitus (%)	55.8%	46.9%	0.059
SAH (%)	67.8%	73.6%	< 0.0001
Smoking (%)	24.2%	30.5%	0.007
FH Positive for CAD (%)	10.1%	13.4%	0.044
Dyslipidemia (%)	48.9%	51.2%	0.292
HF (%)	10.7%	8.8%	0.039
Previous stroke (%)	5.4%	4.9%	0.073
Previous AMI (%)	40.3%	36.8%	0.091
Previous CABS (%)	18.2%	16.0%	0.607
Previous CA (%)	22.7%	23.2%	0.773
Hb (%) (mean)	42.7 ± 2.31	41.1 ± 2.48	0.24
Peak troponin (mean) (ng/dL)	13.2 ± 3.21	11.8 ± 4.37	0.32
Cr (mg/dL) (mean)	1.25 ± 0.54	1.52 ± 0.67	0.168
SBP (mmHg) (média)	132.1 ± 26.86	132.3 ± 24.53	0.636
LVEF (%) (média)	56% + 13.4%	52.1% + 11.8%	0.586
Killip ≥ 2 (%)	1.8%	5.6%	0.003
ASA (%)	98.5%	97.8%	0.87
Beta-blocker (%)	96.1%	87.4%	< 0.0001
Clopidogrel (%)	65.4%	67.9%	0.038
GP Iib/IIIa inhibitor (%)	5.8%	16.1%	< 0.0001
ACEI (%)	74.3%	69.2%	0.06
Statin (%)	98.5%	93.8%	< 0.0001

SBP: systolic blood pressure; SAH: systemic arterial hypertension; FH: Family history; CAD: coronary artery disease; HF: heart failure; AMI: acute myocardial infarction; CABS: coronary artery bypass surgery; CA: coronary angioplasty; Hb: hemoglobin; CR: creatinine; LVEF: left ventricle ejection fraction; GP: glycoprotein inhibitor; ACEI: angiotensin converting enzyme inhibitor.

All calculations were done using the software SPSS v10.0.

## Results

Mean age was 61 years old, and approximately 63% of participants were male. The most prevalent risk factor was systemic arterial hypertension, in 71% of cases. Mean Mehran bleeding score was 16.2 versus 15.7 in fondaparinux and enoxaparin groups, respectively. In relation to treatment, we observed percutaneous coronary intervention in 40.2% in the fondaparinux group, and 35.1% in the enoxaparin group (p = 0.13). Coronary artery bypass surgery was done in 18.8% of the fondaparinux group versus 17.6% of the enoxaparin group (p = 0.031). In relation to the coronary arterial pattern, no significant differences were observed between the groups fondaparinux and enoxaparin, with 45.2% versus 43.6% one-vessel (p = 0.432), 20.1% versus 17.9% two-vessel (p = 0.567), and 22.3% versus 24.9% three-vessel (p = 0.871), respectively.

With regards to the occurrence of haemorrhagic complications, femoral artery pseudoaneurysm was the most frequent (56% of cases), followed by hemorrhagic stroke (18%) and high digestive bleeding associated to hemodynamic instability and/or drop in hemoglobin ≥ 3,0 g/dL (16%). No significant differences were observed between the types of bleeding related to enoxaparin versus fondaparinux.

In the comparison between the groups, significant differences were observed in relation to hypertension (67.8% vs. 73.6%, p < 0.0001); smoking (24.2% vs. 30.5%, p = 0.007); family history of early onset coronary disease (10.1% vs. 13.4%, p = 0.044); heart failure (10.7% vs. 8.8%, p = 0.039). killip classification > 2 2 (1.8% vs. 5.6%, p = 0.003); use of beta-blockers (96.1% vs. 87.4%, p < 0.0001); clopidogrel (65.4% vs. 67.9%, p < 0.038); glycoprotein IIbIIIa inhibitor (5.8% vs. 16.1%, p < 0.0001); and statins (98.5% vs. 93.8%, p < 0.0001). Basal characteristics of the studied population are depicted in Table 1.

**Figure 1:**
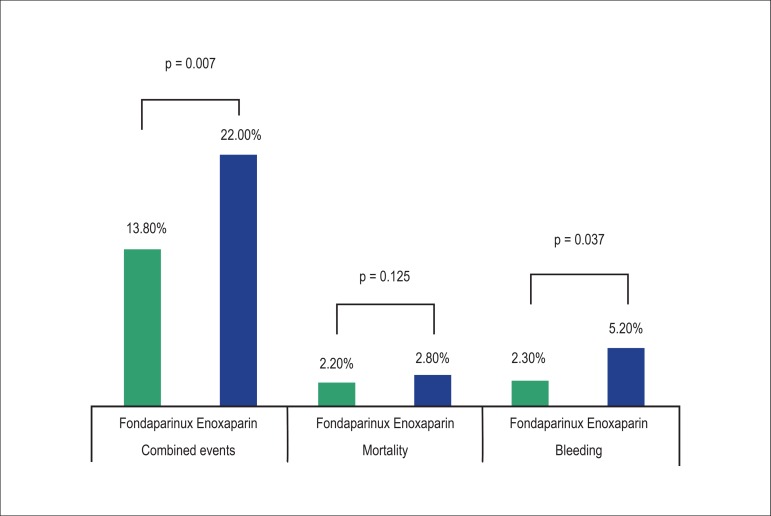
Comparative evaluation of mortality, combined events and bleeding between the groups fondaparinux and enoxaparin.

In the multivariate analysis, significant differences were observed between the fondaparinux and enoxaparin groups in relation to combined events (13.8% vs. 22%, OR = 2.93, p = 0.007) and bleeding (2.3% vs. 5.2%, OR = 4.55, p = 0.037), respectively. Multivariate analysis results comparing different in-hospital outcomes between the groups are presented in Table 2 and Figure 1.

**Table 2:** Multivariate analysis results comparing different in-hospital outcomes between the groups of patients on fondaparinux versus enoxaparin

	Fondaparinux	Enoxaparin	OR	CI 95%	p
Reinfarction	6.1%	10.5%	1.23	0.27 - 5.62	0.7
Cardiogenic shock	2.1%	2.9%	6.38	0.80 - 50.78	0.08
Bleeding	2.3%	5.2%	4.55	1.09 - 18.91	0.037
Stroke	1.1%	0.6%	2.49	0.32 - 7.85	0.376
Mortality	2.2%	2.8%	1.71	0.49 - 5.93	0.125
Combined events	13.8%	22.0%	2.93	1.34 - 6.42	0.007

OR: Odds ratio; CI: confidence intervals.

## Discussion

The present study showed important data reproduced in the Brazilian population that are in line with results from recent publications from literature. We observed a significant reduction of bleeding and combined events during in-hospital evolution. With regards to mortality, no significant difference was found between fondaparinux and enoxaparin patients.

In 2006, the study OASIS-5 was published, which was a randomized work with 20,078 patients with NSTEACS that received 2.5 mg fondaparinux versus 1 mg/kg enoxaparin twice per day, effectively comparing the two anticoagulants. Similar results were observed as far as the primary outcome of the study in relation to combined events during hospital stay (death and reinfarction). However, after nine days, the highest rates of bleeding with fondaparinux use were significantly reduced in comparison to patients who received enoxaparin (2.2% vs. 4.1%, p < 0.001). Moreover, fondaparinux kept its superiority in relation to long-term bleeding and proved to be better in relation to 30-day mortality (2.9% vs. 3.5%, p = 0.02) and 180-day mortality (5.8% vs. 6.5%, p = 0.05).^2,4,9^

After the main study was published, there was still some doubt on whether the same results could be reproduced in the general population, with no specific selection criteria. However, fondaparinux use has considerably expanded, especially in Europe, becoming an Ib indication by the European Society of Cardiology in patients with NSTEACS, whereas enoxaparin remained an Ib indication through the same guidelines.^10^ Thus, some data banks were published, showing similar results to OASIS-5, but in real life.^3,5,11,12^

Of all registries, the most impactful was the Swedish registry comparing fondaparinux to enoxaparin in approximately 40,000 patients with NSTEACS. Around 36.4% of those were treated with fondaparinux, and 63.6% with enoxaparin. Lower bleeding rates were observed comparatively between fondaparinux and enoxaparin (1.1% vs. 1.8%, OR = 0.54, CI 95% = 0.42 - 070). This was also reflected in lower in-hospital mortality rates in patients who received fondaparinux (2.7% vs. 4.0%, OR = 0.75, CI 95% = 0.63 - 0.89). After 30 and 180 days, differences related to mortality and bleeding were maintained between the groups. Such finding reflected, partially, what the OASIS-5 study had demonstrated, except this time, in a real population from a significant sample.^5^ This way, our study results are in line with what literature has been presenting, showing lower bleeding and combined event rates.

Undoubtedly, the main differentiator between fondaparinux and enoxaparin is the lower risk of bleeding associated with its use. Even when there is percutaneous coronary intervention, or when it is associated to the use of glycoprotein IIbIIIa inhibitors, fondaparinux shows lower bleeding rates in comparison to enoxaparin.^13,14^ In 2009, Budaj et al.^15^ published an OASIS-5 study subanalysis, showing that this reduction happens in almost all types of bleeding when fondaparinux is used, with the exception of intracranial bleeding and bleeding related to surgeries, where no difference is found. Moreover, justifying the importance of bleeding in patient evolution and its correlation to other outcomes, the authors showed a mortality of 8.4% vs. 2.7% (p < 0.0001), respectively, between patients who presented, or not, major bleeding.^16^ Even though we did not show, in our study, significant differences in relation to mortality, bleeding increase resulted in a higher number of combined events.

The justification for the lower bleeding rate is partly due to the use of one reduced anticoagulant dose when fondaparinux is administered. However, such dose of 2.5 mg per day was previously validated, showing that in the duration of a dual antiplatelet therapy, the required anticoagulant dose for a complete system block should possibly be reduced. Additionally, fondaparinux is a very specific and reversible factor Xa inhibitor, which means that, in theory, a smaller dose is amplified in terms of the anticoagulant effect.^1^

Lastly, due to bleeding reduction and the consequent smaller rate of mortality and events stemming from fondaparinux use, several studies have shown better cost-benefit of its use in relation to enoxaparin.^16-19^ An OASIS-5 study subanalysis showed, after 180 days, an average cost reduction of up to 547 dollars per patient in the group that used fondaparinux, highlighting the medication's superiority even further.^16^

Thus, fondaparinux use in NSTEACS patients has been expanding in Brazil and worldwide. In this scenario, a demonstration of the same benefit in a Brazilian registry is pivotal to give more security and reliability to the country.

### Limitations

Despite our large sample, this is a retrospective study, and it presents a much larger number of patients on enoxaparin than on fondaparinux, We believe that such differences are based on attending physicians' longer experience with patients on enoxaparin, especially since this medication has been in use for longer by the Brazilian population when compared to fondaparinux. Moreover, we do not have the description of the type of vascular access that was used, which can influence the bleeding rate associated to percutaneous coronary intervention. Percutaneous coronary intervention rate is considered relatively low, probably due to high complexity profile of patients involved in the study. Lastly, the use of glycoprotein IIbIIIa inhibitors was higher in the enoxaparin group, which may, partially, increase the bleeding rate in this group.

## Conclusion

Similarly to the recently published data in international literature, fondaparinux was proved superior to enoxaparin when administered in the Brazilian population, with significant reduction of combined events and bleeding.

## References

[1] Simoons ML, Bobbink IW, Boland J, Gardien M, Klootwijk P, Lensing AW, PENTUA Investigators (2004). A dose-finding study of fondaparinux in patients with
non-ST-segment elevation acute coronary syndromes: the Pentasaccharide in
Unstable Angina (PENTUA) Study. J Am Coll Cardiol.

[2] Schiele F (2010). Fondaparinux and acute coronary syndromes: update on the OASIS
5-6 studies. Vasc Health Risk Manag.

[3] Schiele F, Meneveau N, Seronde MF, Descotes-Genon V, Dutheil J, Chopard R, Reseau de Cardiologie de Franche Comte (2010). Reseau de Cardiologie de Franche Comte. Routine use of
fondaparinux in acute coronary syndromes: a 2-year multicenter
experience. Am Heart J.

[4] Yusuf S, Mehta SR, Chrolavicius S, Afzal R, Pogue J, Granger CB, Fifth Organization to Assess Strategies in Acute Ischemic Syndromes
Investigators (2006). Comparison of fondaparinux and enoxaparin in acute coronary
syndromes. N Engl J Med.

[5] Szummer K, Oldgren J, Lindhagen L, Carrero JJ, Evans M, Spaak J (2015). Association between the use of fondaparinux vs
low-molecular-weight heparin and clinical outcomes in patients with
non-ST-segment elevation myocardial infarction. JAMA.

[6] Nicolau JC, Timerman A, Marin-Neto JA, Piegas LS, Barbosa CJ, Franci A, Sociedade Brasileira de Cardiologia (2014). Guidelines of Sociedade Brasileira de Cardiologia for Unstable
Angina and Non-ST-Segment Elevation Myocardial Infarction (II Edition, 2007)
2013-2014 Update. Arq Bras Cardiol.

[7] Jneid H, Anderson JL, Wright RS, Adams CD, Bridges CR, Casey DE, American College of Cardiology Foundation, American Heart Association Task Force on Practice
Guidelines (2012). 2012 ACCF/AHA focused update of the guideline for the management
of patients with unstable angina/non-ST-elevation myocardial infarction
(updating the 2007 guideline and replacing the 2011 focused update): a
report of the American College of Cardiology Foundation/American Heart
Association Task Force on practice guidelines. Circulation.

[8] Mehran R, Rao SV, Bahht DL, Gibson M, Caixeta A, Eikelboom J (2011). Standardized bleeding definitions for cardiovascular clinical
trials: a consensus report from the Bleeding Academic Research
Consortium. Circulation.

[9] Majure DT, Aberegg SK (2006). Fondaparinux versus enoxaparin in acute coronary
syndromes. N Engl J Med.

[10] Roffi M, Patrono C, Collet J, Mueller C, Valgimigli M, Andreotti F (2016). Management of Acute Coronary Syndromes in Patients Presenting
without Persistent ST-Segment Elevation of the European Society of
Cardiology. 2015 ESC guidelines for the management of acute coronary
syndromes in patients presenting without persistent ST-segment elevation:
Task Force for the Management of Acute Coronary Syndromes in Patients
Presenting without Persistent ST-Segment Elevation of the European Society
of Cardiology (ESC). Eur Heart J.

[11] Permsuwan U, Chaiyakunapruk N, Nathisuwan S, Sukonthasarn A (2015). Cost-effectiveness analysis of fondaparinux vs enoxaparin in
non-ST elevation acute coronary syndrome in Thailand. Heart Lung Circ.

[12] Kossovsky M, Keller PF, Mach F, Gaspoz JM (2012). Fondaparinux versus enoxaprin in the management of acute coronary
syndromes in Switzerland: a cost comparison analysis. Swiss Med Wkly.

[13] Mehta SR, Granger CB, Eikelboom JW, Bassand JP, Wallentin L, Faxon DP (2007). Efficacy and safety of fondaparinux versus enoxaparin in patients
with acute coronary syndromes undergoing percutaneous coronary intervention:
results from the OASIS-5 trial. J Am Coll Cardiol.

[14] Jolly SS, Faxon DP, Fox KA, Afzal R, Boden WE, Widimsky P (2009). Efficacy and safety of fondaparinux versus enoxaparin in patients
with acute coronary syndromes treated with glycoprotein IIb/IIIa inhibitors
or thienopyridines: results from the OASIS 5 (Fifth Organization to Assess
Strategies in Ischemic Syndromes) trial. J Am Coll Cardiol.

[15] Budaj A, Eikelboom JW, Mehta SR, Afzal R, Chrolavicius S, Bassand JP, OASIS 5 Investigators (2009). Improving clinical outcomes by reducing bleeding in patients with
non-ST-elevation acute coronary syndromes. Eur Heart J.

[16] Sculpher MJ, Lozano-Ortega G, Sambrook J, Palmer S, Ormanidhi O, Bakhai A (2009). Fondaparinux versus Enoxaparin in non-ST-elevation acute coronary
syndromes: short-term cost and long-term cost-effectiveness using data from
the Fifth Organization to Assess Strategies in Acute Ischemic Syndromes
Investigators (OASIS-5) trial. Am Heart J.

[17] Huber K, Bates ER, Valgimigli M, Wallentin L, Kristensen SD, Anderson JL (2014). Antiplatelet and anticoagulation agents in acute coronary
syndromes: what is the current status and what does the future
hold?. Am Heart J.

[18] Ross Terres JA, Lozano-Ortega G, Kendall R, Sculpher MJ (2015). Cost-effectiveness of fondaparinux versus enoxaparin in
non-ST-elevation acute coronary syndrome in Canada (OASIS-5). BMC Cardiovasc Disord.

[19] Pepe C, Machado M, Olimpio A, Ramos R (2012). Cost-effectiveness of fondaparinux in patients with acute
coronary syndrome without ST-segment elevation. Arq Bras Cardiol.

